# Treatment patterns and characteristics of European patients with castration-resistant prostate cancer

**DOI:** 10.1186/1471-2490-13-58

**Published:** 2013-11-09

**Authors:** Cora N Sternberg, Edwina S Baskin-Bey, Mark Watson, Andrew Worsfold, Alex Rider, Bertrand Tombal

**Affiliations:** 1 San Camillo and Forlanini Hospitals, Rome, Italy; 2 Astellas Pharma Europe, Leiden, Netherlands; 3 Adelphi Real World, Macclesfield, UK; 4 Cliniques universitaires Saint-Luc UCL, Institut de Recherche Clinique, Université catholique de Louvain, Brussels, Belgium; 5 Department of Medical Oncology, San Camillo Forlanini Hospital, Padiglione Flajani, 1st floor, Circonvallazione Gianicolense 87, Rome 00152, Italy

**Keywords:** Castrate-resistant prostate cancer, CRPC, treatment, hormonal therapy, Anti-androgen, LHRH, Chemotherapy, Survey

## Abstract

**Background:**

European treatment guidelines recommend the use of hormonal therapy for the treatment of advanced prostate cancer, including castration-resistant prostate cancer (CRPC), but there is little understanding of how common practices in prostate cancer treatment compare across Europe. The aim of this analysis was to evaluate the management of CRPC patients across five European countries (France, Germany, Italy, Spain and the UK).

**Methods:**

Data were drawn from the Adelphi Real World Prostate Cancer Disease Specific Programme (DSP), a cross-sectional survey of patients undertaken between December 2009 and May 2010. The study is based on physician interviews, physician-completed detailed patient record forms, and a patient-completed questionnaire.

**Results:**

A total of 348 physicians (191 urologists and 157 oncologists) reported on 3477 patients with prostate cancer. Of the 3477 patients, 1405 (40%) were categorised as having CRPC, and 1119 of these had metastatic CRPC. Bone metastases were the most common (78%), followed by liver (37%) and lung (30%). The mean age of CRPC patients was 71 years, 35% were current or ex-smokers and 10% had a family history of prostate cancer. CRPC patients had a mean of 1.8 comorbidities; 66% had hypertension and 32% had diabetes. Most physicians estimated their patients would stop responding to initial hormone therapy after 19–24 months. Overall, addition of an anti-androgen to a luteinising-hormone-releasing hormone (LHRH) agonist was the most commonly prescribed therapy when patients failed initial LHRH agonist therapy, although there were considerable variations between countries. While 72% of physicians in Europe would choose chemotherapy as the next treatment option after diagnosis of CRPC, 31% of this group would initially prescribe this without an LHRH agonist.

**Conclusions:**

Results from this analysis highlight inconsistencies in common hormonal therapy treatment patterns for CRPC and hormonal therapy across the EU.

## Background

Prostate cancer is the most commonly diagnosed cancer in men [1], with approximately 300,000 new cases diagnosed each year [1] and an annual incidence of 93.1 cases per 100,000 men in Europe (age-standardised to the European population) [2]. Prostate cancer cell growth is dependent on androgens, and evidence shows that androgen blockade can impede the progression of prostate cancer [3]. This can be achieved with surgical castration, or medical castration using androgen-deprivation therapy (ADT). Luteinising-hormone-releasing hormone (LHRH) agonists are the standard of care in ADT [4,5]. However, ADT is not curative, and most prostate cancers progress to an advanced stage known as castration-resistant prostate cancer (CRPC), defined as prostate cancer that progresses despite castrate levels of testosterone (< 50 ng/mL) [4,5].

In Europe, guidelines for the treatment of prostate cancer, including CRPC, have been developed and published by the European Association of Urology (EAU) [4,5], but there are questions regarding adherence to them. EAU guidelines recommend that hormonal therapy remains a cornerstone of treatment of prostate cancer after failure of ADT [4,5]. However, a lack of randomised evidence has been cited as a rationale for withdrawing hormonal therapy from patients who undergo biochemical relapse [6]. The most effective use of ADT in CRPC has thus been a topic of debate, and data from this analysis show that usage continues to be inconsistent across Europe.

The authors undertook an analysis of CRPC patients across Europe, evaluating patient characteristics and treatment patterns of patients with CRPC, the use of ADT in advanced prostate cancer and the management practices of physicians routinely treating CRPC patients.

## Methods

Data were extracted from the Adelphi Real World Prostate Cancer Disease-Specific Programme^©^ (DSP), a cross-sectional survey of 348 urologists and oncologists and their prostate cancer patients conducted between December 2009 and May 2010 in France, Germany, Italy, Spain and the UK. It was performed according to European Pharmaceutical Market Research Association guidelines, and in full accordance with the US Health Insurance Portability and Accountability Act 1996. Each patient provided consent for anonymous and aggregated reporting of research findings as required by the guidelines.

A full description of the methodology has been published previously [7]. Fully de-identified patient and related physician information were provided to and aggregated by Adelphi Real World (Macclesfield, UK), prior to the initiation of the present analysis and author access to the data set.

### Participating physicians

Physicians – urologists and oncologists – were identified by the local DSP fieldwork teams from public lists of healthcare professionals. Physicians were randomly contacted by telephone by local fieldwork personnel and asked a series of screening questions. Candidate physicians who met the eligibility criteria were subsequently invited to participate in the full programme. To be eligible to participate in the study, urologists and oncologists had to have been qualified for ≥3 years and for ≤39 years, to be consulting with at least five prostate cancer patients per week and had to be making the treatment decisions for their patients.

Fieldwork personnel fluent in the local language were responsible for the recruitment of physicians, as well as the collection, collation and audit of all completed DSP materials. All fieldwork agencies in each country were affiliated to a national body that governs data collection procedures and laws in that country, and were subject to annual and/or random audits. To avoid potential selection bias as a result of variable population densities in different geographical regions in a given country, an appropriately larger sample of physicians was identified in densely populated areas than in sparsely populated areas. Recruited physicians were given an incentive to take part in the study, proportionate to their estimated time required to complete the interview, and the required number of patient record forms.

### Patients

All patients with prostate cancer diagnosed by a physician and who were being treated – or who had been actively treated – were eligible for inclusion in the survey. Each physician completed a comprehensive patient record form (PRF) for their two most recently seen CRPC patients and also for their next eight consulting prostate cancer patients receiving active or palliative treatment for their condition. The real-world design of the survey ensured collection only of information available to the physician/patient at the time of consultation. Therefore, no tests or investigations were required or conducted for a patient to be included in the study.

### Data collection

Data collected included: patient characteristics, concomitant conditions and smoking history, date of original diagnosis, symptomatology, Karnofsky performance status at diagnosis, physician-estimated life expectancy, resource utilisation, diagnostic procedures, Gleason score, treatment history and planned treatment changes going forward. Disease status was categorised according to TNM status.

Each physician was interviewed about current management practices and then asked to collect demographic data and clinical and treatment histories for their 10 eligible prostate cancer patients. Of these, eight were invited to complete a patient self-completion questionnaire (PSC), independently of their physician, about health-related quality of life (HRQoL) using the EQ-5D utility score (http://www.euroqol.org). At no stage did physicians see or influence the responses made by their patients, and completion of the PSC was voluntary. A patient’s decision not to complete the questionnaire did not disqualify data recorded by the physician on the PRF from inclusion in the analysis. Matching the physician and patient responses via patient/physician study numbers allowed the PSC data to be linked with comparable data recorded on the PRF to highlight any areas of disparity and/or agreement. Descriptive statistics (e.g. means and proportions) were derived using QPSMR Reflect Version 2007.1 g (QPSMR Ltd., Wallingford, UK).

## Results

### Physician and patient respondents

A total of 1214 physicians were contacted and 348 (29%) participated in the survey. The 71% of physicians that did not participate were either uninterested or unwilling to take part, or did not meet the inclusion criteria described to them during the screening call. Of the total 1214 physicians, 191 (55%) were urologists and 157 (45%) were medical/clinical oncologists (Table 1). Of the urologists, 104 (54%) were practising in academic institutions, 50 (26%) were from a regional/general hospital, 20 (10%) were from private/independent hospitals or practices, 14 (7%) were office-based and 3 (2%) were from specialist cancer units. Of the oncologists, 86 (55%) were practising in academic institutions, 30 (19%) were from a regional/general hospital, 11 (7%) were from private/independent hospitals or practices, 6 (4%) were office-based and 24 (15%) were from specialist cancer units.

**Table 1:** Physician and patient characteristics by country

**Characteristic**	**France**	**Germany**	**Italy**	**Spain**	**UK**	**Total**
Physicians	75	75	72	75	51	348
Urologists	41	41	38	41	30	191
Oncologists	34	34	34	34	21	157
Patient sample size	750	782	720	750	475	3477
Proportion with CRPC	330 (44)	347 (44)	294 (41)	288 (38)	146 (31)	1405 (40)
Proportion with mCRPC	272 (36)	296 (38)	218 (30)	221 (29)	112 (24)	1119 (32)
% of CRPC patients	82	85	74	77	77	80
Disease status						
Localised	170 (23)	172 (22)	219 (30)	171 (23)	105 (22)	837 (24)
Locally advanced	35 (5)	60 (8)	47 (7)	41 (5)	26 (5)	209 (6)
Metastatic	419 (56)	435 (56)	302 (42)	391 (52)	228 (48)	1775 (51)
Unknown	126 (17)	115 (15)	152 (21)	147 (20)	116 (24)	656 (19)
Comorbidities at baseline, mean (SD)	1.8 (1.4)	1.7 (1.4)	1.8 (1.4)	2.2 (1.4)	1.7 (1.3)	1.8 (1.5)
Hypertension, n (%)	777 (65.5)	158 (59.9)	214 (73.3)	191 (70.2)	138 (57.7)	76 (64.4)
Diabetes, n (%)	376 (31.7)	68 (25.8)	103 (35.3)	98 (36.0)	72 (30.1)	35 (29.7)
Hyperlipidaemia, n (%)	338 (28.5)	87 (33.0)	59 (20.2)	82 (30.2)	90 (37.7)	20 (17.0)

Data are mean (%) unless otherwise indicated. mCRPC: metastatic CRPC.

Records for a total of 3477 patients were provided by physicians from all five countries. The PSC was completed by 1236 patients; 2241 patients declined to complete the survey. Statistical comparisons were not performed but a qualitative comparison of patients that completed the PSC (completers) vs those that did not (non-completers) revealed little difference between the two groups in age, Gleason score at diagnosis, body mass index, employment status, smoking status, concomitant conditions, current Karnofsky score, or number of previous and planned consultations with physician. There was a slight tendency for non-completers to have more advanced disease: current staging was metastatic in 54% of non-completers vs 44% of completers; 46% of non-completers had CRPC vs 31% in completers. Overall, 1405 (40%) had CRPC at the time of the study (Table 1), of whom the majority (1119, or 80% of the CRPC patients) had metastatic CRPC.

### CRPC patient profile

Characteristics of CRPC patients showed little variation among countries (Table 2). On average, patients were elderly (mean age 70.8 years), and approximately one-third were smokers. Notably, a family history of prostate cancer was observed in only 10% of patients. The majority of patients had two comorbidities (mean 1.8), with hypertension (65.6%) and diabetes (31.7%) being the most frequently reported (Table 1). Among CRPC patients with metastases, bone metastases were the most common (78% of metastatic patients), followed by liver (37%) and lung (30%). Distributions of metastases between countries were similar, apart from a relatively low incidence of liver metastases in French and UK patients (both 19%) compared with Germany, Italy and Spain (46%, 47% and 32%, respectively).

**Table 2:** CRPC patient characteristics and disease profiles

**Characteristic**	**Total (n = 1405)**
Age, years	70.8 (69.5–71.5)
Time since initial PC diagnosis, years	3.1 (2.6–3.9)
Karnofsky performance score at diagnosis, % patients	
≥50	98%
≥70	89%
≥90	57%
Gleason score	7
Life expectancy, years	73.2 (67.0–72.3)
Current/ex-smokers, %	35 (26–45)
Receiving treatment for current disease, %	36 (26–44)
Family history of prostate cancer, %	10 (8–14)
Body mass index, kg/m^2^	25.8 (25.2–26.5)
Number of comorbidities	1.8 (1.7–2.2)
Surgical procedures, %:	
Radical prostatectomy	28
TURP	12
Pelvic lymph node dissection	7

Data are mean (range) unless otherwise indicated.

### Health status

Overall, the HRQoL of prostate cancer patients was poorer in those with metastatic prostate cancer. Average EQ-5D visual analogue scale (VAS) scores were 74.8, 71.6, 66.3 and 66.5 for patients with localised, locally advanced, metastatic and castrate-resistant prostate cancer, respectively.

### Managing physicians

Urologists co-managed their patients with radiation oncologists in 22% of cases and with medical oncologists in 16% of cases. Of those patients co-managed with medical oncologists, the majority (73%) had metastatic disease. Oncologists co-managed their patients with urologists (in 37% of cases) and with radiation oncologists (in 30% of cases). Of those patients co-managed with urologists, 46% had metastatic disease, while 58% of those co-managed with radiation oncologists had metastatic disease.

The number of years in practice of participating physicians varied between countries; the percentage of physicians practicing for ≤10 years in total was 56% in France, 33% in Germany, 61% in Italy and 55% in Spain, compared with 16%, 40%, 28% and 23%, respectively, practicing for ≥16 years. However, 67% of participating physicians in France, 64% in Germany, 58% in Italy and 76% in Spain had been in their current role for ≤10 years, compared with 11%, 13%, 19% and 13%, respectively, for ≥16 years. Forty three per cent of participating physicians in the UK had been practicing for ≤12 years, but data for years in their current role was not collected.

**Figure 1:**
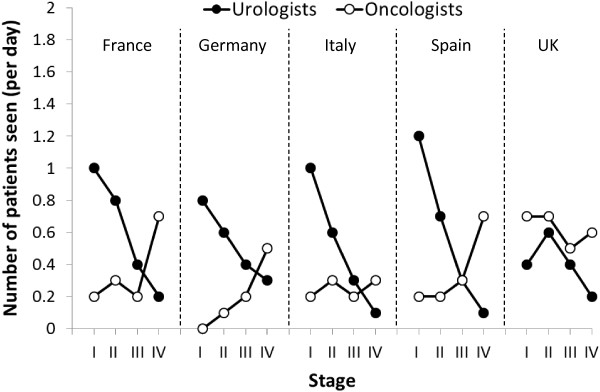
Mean daily number of patients diagnosed <6 months seen by physicians by stage of disease.

Urologists saw an average of 25.6 patients per day (range: 16 [UK] to 37.5 [Germany]), of whom an average of 5.5 had prostate cancer and 2.1 had CRPC. Oncologists saw an average of 21.4 patients per day (range: 12.1 [UK] to 33.7 [Germany]), of whom 3.9 had prostate cancer and 1.3 had CRPC. In general, urologists were more likely to see patients with early-stage disease than with late-stage disease, whereas oncologists were more likely to play a greater part in the management of patients with late stage disease (Figure 1). Exceptions were seen in the UK and Germany. In the UK, urologists and oncologists were involved in patient management to a similar extent across all disease stages except late-stage patients who had been diagnosed with prostate cancer for > 6 months, who were more likely to be managed by oncologists. In Germany, urologists were much more involved in management decisions for patients who had been diagnosed with CRPC for > 6 months and, in contrast to a decrease in involvement in patients diagnosed within 6 months, that involvement increased with disease stage. Overall, 9% of urologists in Europe who would challenge patients with a second hormone treatment stated that they would refer their patient to an oncologist when their prostate cancer progressed to CRPC (France 7%, Germany 2%, Italy 13%, Spain 20% and UK 0%). These figures reflect the earlier involvement of oncologists in some countries, particularly the UK, which removes the need for referral.

### Patient management approaches

**Figure 2:**
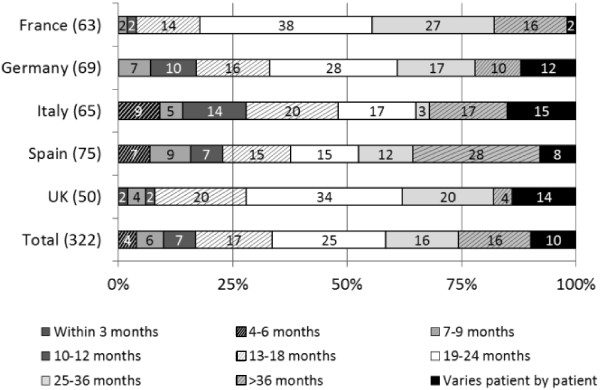
Mean physician-stated time until prostate cancer progresses despite ADT use.

Treatment decisions were found to be similar between countries. A majority of physicians (62% of urologists and 67% of oncologists) stated that they would challenge patients with a second hormonal therapy after prostate cancer progression following initial ADT. This course of action was more likely to be taken by physicians in the UK (80% of urologists and 90% of oncologists) but less likely by German oncologists or Spanish urologists (47% and 39%, respectively). The average time to second hormonal therapy was estimated as being 19–24 months by the majority of physicians in France, Germany and the UK (38%, 28% and 34% respectively), whereas in Spain, 28% of physicians estimated >36 months and in Italy, the main responses varied from between 13 and >36 months (Figure 2).

Of 115 physicians who initially treated with an LHRH agonist, 60 (53%) said they would continue the patient on their initial LHRH agonist therapy after failure of initial LHRH agonist (as defined by elevated PSA) but would add another therapy, whereas 23 (20%) stated they would switch the patient to a different LHRH agonist (Table 3). Thirty two (28%) physicians would discontinue LHRH: 28 (24%) would switch the patient to a different hormonal monotherapy (22 anti-androgen, 6 oestrogen), 2 (2%) would switch to chemotherapy alone and 2 (2%) would switch to monoclonal antibodies alone. Although addition of an anti-androgen was the most common overall choice of secondary therapy, uptake ranged from 21% in Spain to 71% in Italy, while switch to an anti-androgen alone ranged from 0% in Italy to 50% in Spain (Table 3).

**Table 3:** Physician-stated choices for management of prostate cancer patients who no longer respond to their first LHRH agonist

**Therapy prescribed**	**France (n = 23)**	**Germany (n = 31)**	**Italy (n = 17)**	**Spain (n = 14)**	**UK (n = 30)**	**Total (n = 115)**
LHRH agonist + anti-androgen	8 (35)	13 (42)	12 (71)	3 (21)	21 (70)	57 (50)
LHRH agonist alone	6 (26)	9 (29)	4 (24)	2 (14)	2 (7)	23 (20)
Anti-androgen alone	3 (13)	7 (23)	0 (0)	7 (50)	5 (17)	22 (19)
Other*	6 (26)	2 (6)	1 (6)	2 (14)	2 (7)	13 (11)

Data are n (% of physicians).

*Oestrogen, other non-specified hormone therapy, chemotherapy, monoclonal antibody.

**Figure 3:**
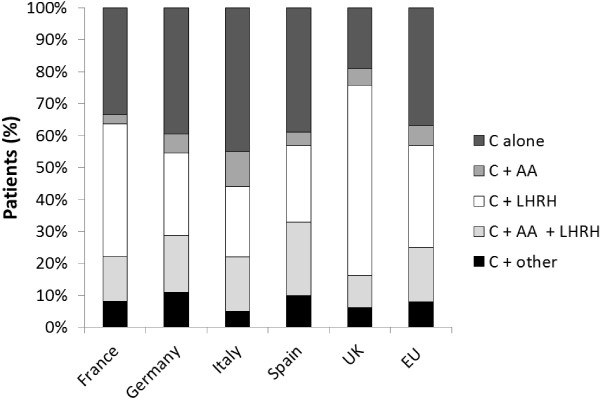
Treatment patterns for patients receiving their first chemotherapy.

PSA was cited as a principal marker of whether a patient was diagnosed with CRPC – either doubling time, rising PSA level or velocity. Castrate testosterone levels were cited by 12% of urologists and 6% of oncologists as an additional marker to help define CRPC. Once diagnosed with CRPC, the majority of patients continued to receive LHRH agonists (85%, 83%, 90%, 88% and 96% in France, Germany, Italy, Spain and the UK, respectively). Overall, 72% of physicians (79% of oncologists and 64% of urologists) stated they would choose chemotherapy as the next treatment option when prostate cancer progression was observed after hormonal therapy in CRPC patients. In this study, 841 (60%) patients determined to have CRPC were selected for chemotherapy either as monotherapy (37%), in combination with an LHRH agonist (32%), an anti-androgen (6%), both an LHRH agonist and an anti-androgen (17%), or another agent (8%). There were some differences between countries: chemotherapy alone was least likely to be prescribed in the UK (19% of patients vs 33–45% in other EU countries), while chemotherapy with an LHRH agonist was the most common option in the UK (59% of patients) (Figure 3).

## Discussion

The results of this physician and patient survey highlight some of the areas of consensus in the patient profiles and treatment approaches for CRPC in Europe. Key areas of divergence between countries are patient management and continued use of LHRH agonists in CRPC patients who progress while on this class of primary hormonal therapy, including those receiving chemotherapy.

Across the countries included in this survey, the data showed that, in general, urologists are more likely to manage earlier-stage prostate cancer, while oncologists are more involved in later-stage disease. However, Germany and the UK are notable exceptions: in Germany, urologists are involved in the full spectrum of disease, while in the UK, it is known that oncologists have a greater role in earlier-stage disease when compared to other countries, with continuing support from urologists after progression to metastatic disease and CRPC. This may explain the finding that no UK physicians of those included in the survey would refer patients to oncologists at the diagnosis of CRPC, as oncologists are already involved. In France, a multidisciplinary approach to patient care is encouraged [8], although this does not appear to be reflected by the results of this survey. Urologist and oncologist case-loads were similar across all five countries, although in the UK the number of patients per physician was notably lower than in other countries.

The survey revealed some trends among countries in treatment decisions for patients with evidence of disease progression. For CRPC patients who progressed while on hormonal therapy, physicians participating in this survey were likely to try an alternative hormonal therapy, either within the same drug class (from one LHRH agonist to another LHRH agonist) or by adding to the underlying LHRH agonist another hormonal therapy from a different drug class. This is consistent with recent evidence suggesting that CRPC remains dependent on AR signalling, and that continued hormonal manipulation may produce clinically relevant responses [5,9-13]. Thus, maintenance of the castrate state is an essential component in the treatment of patients who progress while on hormonal therapy [13]. However, despite the shift in treatment practice towards maintaining LHRH agonists during chemotherapy [4,5,13-16], this survey revealed that patients in Italy, Germany, Spain and France were more likely to receive chemotherapy alone than patients in the UK. The proportion of physicians who had been practicing in their current role for ≤10 years was similar in France, Germany, Italy and Spain, suggesting no clear relationship between number of years practicing and treatment decisions. There were limitations to the data set, and therefore the conclusions that can be reached, given that the number of years a participating physician had practiced in their current role was not collected in the UK and the age of physicians was not collected in all countries.

## Conclusions

As the disease progresses, maintenance of ADT in patients with CRPC is recommended [4,5] However, our data indicate that this guidance may not be followed consistently across France, Germany, Italy, Spain and the UK.

## Abbreviations

ADT: Androgen deprivation therapy; CRPC: Castration-resistant prostate cancer; DSP: Disease-specific programme; HRQoL: Health-related quality of life; LHRH: Luteinising-hormone-releasing hormone; PRFV: Patient record form; PSA: Prostate-specific antigen; PSC: Patient self-completion questionnaire.

## Competing interests

Edwina Baskin-Bey and Mark Watson are employees of Astellas Pharma.

## Authors’ contributions

AR and AW were principal in the conception, design and coordination of this independent real-world study and performed all descriptive data analyses. EBB was involved in data acquisition, analysis and interpretation. All authors helped to draft the manuscript. All authors read and approved the final manuscript.

## Pre-publication history

The pre-publication history for this paper can be accessed here:

http://www.biomedcentral.com/1471-2490/13/58/prepub
